# Prognostic value of systemic immune-inflammation index and systemic inflammation response index for oral cancers: A systematic review and meta-analysis

**DOI:** 10.4317/medoral.26779

**Published:** 2024-10-13

**Authors:** Shaowei Yang, Chenyan Fei

**Affiliations:** 1 Department of Stomatology, The second affiliated hospital of Jiaxing university, Jiaxing City, Zhejiang Province, China

## Abstract

**Background:**

The systemic immune-inflammation index (SII) and systemic inflammation response index (SIRI) are commonly used prognostic indicators for a variety of cancers. However, their utility in oral cancers is unknown. We systematically examined evidence on the ability of SII and SIRI to predict overall survival (OS) and disease-free survival (DFS) after oral cancers.

**Material and Methods:**

Embase, PubMed, Web of Science, and Scopus were searched for oral cancer studies reporting OS or DFS based on SII or SIRI. Articles published up to 25th May 2024 were included.

**Results:**

17 studies were eligible (14 on SII and 3 on SIRI). Pretreatment high SII scores were found to be significantly linked with poor OS (HR: 1.62 95% 1.26, 2.08 I2=88%) and DFS (HR: 1.62 95% 1.25, 2.27 I2=86%) after oral cancer. Similarly, high SIRI was associated with worse OS in oral cancer patients (HR: 1.60 95% 1.31, 1.94 I2=0%). All results were unchanged on sensitivity analysis. Subgroup analysis based on location, cancer type, sample size, treatment, cut-off, methods of determining cut-off, analysis method, and study quality showed mixed results.

**Conclusions:**

Acknowledging the limitations of current evidence, it seems that both SII and SIRI can predict the prognosis of oral cancers. High SII and SIRI are both associated with worse OS while high SII also predicts worse DFS.

** Key words:**Oral carcinoma, inflammation, survival, prognosis.

## Introduction

Oral cancer is one of the commonest malignancies of the head and neck and includes cancer of the lips and oral cavity (tongue, gingiva, floor of the mouth, palate, and other parts of the mouth). It is the 13th most diagnosed cancer worldwide leading to about 377,713 new cases and 177,757 deaths in 2020 alone (1). Significant heterogeneity exists in the distribution of oral cancer with the highest number of cases diagnosed in Southeast Asia and India alone accounting for about 1/3rd of the total global burden of oral cancer (2). The symptoms and the treatment of oral cancer have a major effect on a patient's quality of life affecting both cosmetic appearance and psychological well-being (3). Prognosis remains poor and 5-year survival in developed countries is about 64% (4). In developing countries which have a major share of cases, survival dips further and is only about 50% after 5-years (5). Considering such a poor prognosis, there is a need for obtaining high-quality predictive markers that can accurately assess the prognosis of oral cancer patients thereby helping clinicians in risk stratification.

The importance of systemic inflammation in cancer development and progression is now well studied (6). Long-term infections, immune disorders, or deranged healing at sites of recurrent tissue injury like in the oral cavity can activate cellular pathways leading to initial tumor development and cancer progression (6). Further, chronic inflammation is known to increase tumor cell growth, vascular proliferation, and metastasis thereby leading to worse prognosis (7). Several immuno-inflammatory processes have been implicated in the development of precancerous conditions like oral submucous fibrosis and to a lesser extent lichen planus which can transform into frank oral cancer (6). In this context, quantifying the inflammatory status of the patient can help in predicting prognosis. However, the identification of such a singular marker continues to be a dilemma.

A number of inflammatory markers like neutrophil-lymphocyte ratio, platelet-lymphocyte ratio, albumin, C-reactive protein, pan immune inflammation value, Glasgow Prognostic Score, systemic immune-inflammation index (SII) and systemic inflammation response index (SIRI) have been used to predict cancer prognosis but there is no consensus on which is the best marker (8). Amongst these, SII and SIRI have developed considerable interest as they can be easily calculated from commonly obtained hematological values. SII is generated by multiplying the absolute platelet and neutrophil counts and then dividing it by the absolute lymphocyte count (9). On the other hand, SIRI is calculated with the following formula: neutrophil count × monocyte count/lymphocyte count (10). Both SII (11-13) and SIRI (14-16) are independent predictors of outcomes in several different malignancies. However, their utility for oral cancer remains unclear. We, therefore, conducted this current systematic review and meta-analysis to examine the prognostic ability of SII and SIRI for oral cancers.

## Material and Methods

The PRISMA statement guidelines were followed during the review (17). The review protocol was uploaded to the PROSPERO before beginning the review (CRD42024548725).

- Information sources

An online search was conducted by two reviewers (SY & CF) involving the repositories of Embase, PubMed, Web of Science, and Scopus. The search included all articles published up to 25th May 2024. We did not apply any filters for language and date of publication. The search was restricted to human studies published as full-texts only.

With the aid of a medical librarian experienced in conducting systematic reviews, we utilized several free and MeSH keywords to formulate the following search strategy. Details are shown in Supplement 1. The first query used was: ((systemic immune-inflammation index) OR (systemic inflammation response index)) AND ((((((((oral) OR (mouth)) OR (lip)) OR (buccal)) OR (tongue)) OR (alveolus)) OR (palatal)) AND (cancer)). The second query used was: ((systemic immune-inflammation index) OR (systemic inflammation response index)) AND (oral squamous cell carcinoma). To complement the database search, we also scrutinized Google Scholar for gray literature. Additionally, the bibliography of included articles were also screened.

- Study selection

A three-step protocol was carried out for the selection of studies. In the first step, all search results were combined and duplicated studies were deleted. In the second step, two reviewers independently scrutinized the unique articles by reading their titles and abstracts. Important studies were selected and downloaded for step three. In the third step, the same reviewers conducted the final selection of studies by reading the full texts. Any disagreement was resolved through consensus.

- PECOTS eligibility criteria

We used the following PECOTS framework to screen studies for this review:

Population: Studies conducted on adult oral cancer patients. Types of oral cancer included were: lip, buccal mucosa, tongue, gingival, floor of the mouth, and palate.

Exposure: High SII or SIRI

Comparison: Low SII or SIRI

Outcome: Clinico-pathological parameters, overall survival (OS) or disease-free survival (DFS)

Time: Any follow-up duration

Study type: primary-level observational studies

- Inclusion criteria

We considered studies with the following criteria:

1. Cohort or case-control study designs.

2. Studies conducted on patients with any type of oral cancer.

3. Studies examining the association between high vs low SII or SIRI and OS or DFS.

3. Studies reporting OS and DFS.

4. Studies reporting the effect size of the association with 95% confidence intervals (CI) in a univariate or multivariate analysis. If both were reported, the latter was preferred.

The investigations that met the following criteria were excluded:

1. Studies not reporting outcome data in numerical form with 95% CI.

2. Studies on head and neck cancers and not reported separately for oral cancers.

3. Review articles, meta-analyses, abstracts, and commentaries.

- Risk of bias

The quality was judged by the QUIPS-Quality in Prognosis Studies tool (18). Two reviewers (SY & CF) conducted the risk of bias analysis with disagreements being resolved by consensus. Studies were judged for the following domains: study participation, study attrition, prognostic factor measurement, outcome assessment, measurement of and controlling for confounding variables, and statistical analysis.

- Data management

Two reviewers (SY & CF) used a pre-piloted Table for extracting study-related information. Information extracted was: first author name, year of publication, location, study design, sample size, median age, male gender, tumor stage, treatment, SII/SIRI cut-off, method of determining cut-off, outcomes, and follow-up. The primary outcome was OS whereas the secondary outcome was DFS.

- Statistical analysis

The primary aim was to examine the relative risks of OS and DFS of oral cancer patients with high vs low SII and SIRI. This was presented as hazard ratios (HRs) and confidence intervals (CIs). We used “Review Manager” (RevMan, version 5.3) to perform the meta-analysis. HR>1 demonstrated worse OS and DFS. We did not extract data from Kaplan Meier curves or from raw dataset of the studies and only directly reported values were used. Cochrane’s Q-test and I2 statistics determined the study heterogeneity, and the significant heterogeneity was estimated by an I2 > 50 %. The effect sizes of studies were combined using a random-effects model. Publication bias was examined by assessing the symmetry of funnel plots and Egger’s test. Significance for Egger’s test was set as *p*<0.10. A sensitivity analysis involving the removal of one study at a time was conducted to assess the credibility of the results. Subgroup analysis was conducted based on location, cancer type, sample size, treatment, cut-off, methods of determining cut-off, analysis method, and risk of bias.

## Results

- Search results

**Figure 1:**
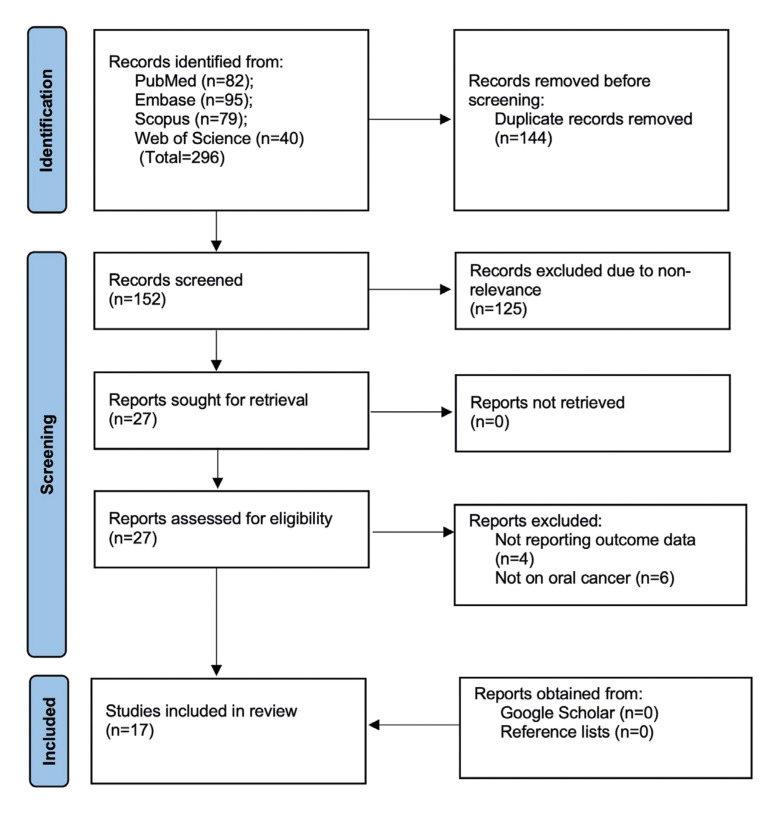
Study flowchart.

Two hundred and ninety six studies were found in the database search. No additional study was found from Google Scholar or reference lists. After deduplication, 152 studies underwent screening, and 27 were chosen for complete text analysis. Finally, 17 fulfilled the inclusion criteria and were selected (9,10,19-33) (Fig. 1). There was 100% agreement between the two reviewers for selection of studies with kappa=1.

- Baseline details

All included studies were retrospective cohort studies (Table 1). There were 14 studies on SII and three studies corresponding to four cohorts on SIRI. No study reported both SII and SIRI. SII and SIRI were calculated pretreatment in all studies. The included studies were recently published between the years 2018 to 2024. The location of the studies was mostly China (8 studies). The remaining were from Taiwan, Korea, Japan, Malaysia, Turkey, Brazil and Spain.

In the case of SII, most studies included patients with unspecified oral squamous cell carcinoma (OSCC), three included only tongue cancer while one study included only lip cancer. The 14 studies included a combined total of 4508 patients. The median age of patients was mostly above 50 years of age. There was one study that included only stage III-IV cancer while all other studies included all cancer stages. Most studies used the receiver operating characteristic (ROC) curve to calculate the cut-off of SII while three studies used the X-tile software and one used the cut-off based on literature. The cut-off ranged from 204 to 1137. Seven studies used multivariate analysis to examine the effect of SII on outcomes while five studies reported only univariate analysis. Treatment involved surgery with or without chemotherapy and radiotherapy in all studies except for one study where only radiotherapy was used. The NOS scores of the studies were found to vary between six and eight.

Amongst the three studies on SIRI, two were from China, one was from Spain. All were on unspecified OSCC. The cumulative sample size of the cohorts was 2258. Median age of patients was >50 years in all four cohorts. One study included only stage I-II cancer while three cohorts included all stages. One study treated patients with only surgery while surgery was combined with chemotherapy and radiotherapy in three cohorts. The cut-off of SIRI ranged from 1 to 1.3. All studies reported only on OS. Three cohorts used multivariate analysis while one used only univariate analysis. Risk of bias analysis based on QUIPS-Quality in Prognosis Studies tool is presented in Table 2. All studies had moderate to high risk of bias.

- Meta-analysis

**Figure 2:**
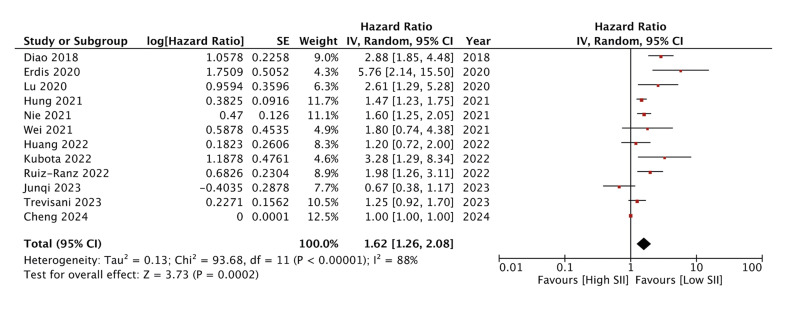
Meta-analysis examining the association between SII and OS after oral cancer.

**Figure 3:**
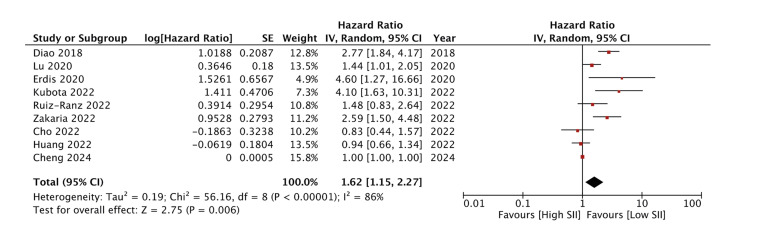
Meta-analysis examining the association between SII and DFS after oral cancer.

Meta-analysis on the link between SII and OS in oral cancer patients with data from 12 studies is shown in Fig. 2. High SII scores were found to be significantly associated with worse OS (HR: 1.62 95% 1.26, 2.08 I2=88%). Q test also showed high heterogeneity (*p*<0.00001). The reviewers did not note any major asymmetry on the funnel plot to indicate publication bias (Supplement 2). Egger’s test indicated non-significant results (*p*=0.19). Similarly, a pooled analysis of nine studies examining the association between SIRI and DFS is depicted in Fig. 3.

High SII was found to worsen DFS (HR: 1.62 95% 1.25, 2.27 I2=86%). Q test also showed high heterogeneity (*p*<0.00001). Here too, the authors did not note any publication bias on the funnel plot or on Egger’s test (*p*=0.11) (Supplement 3).

**Figure 4:**
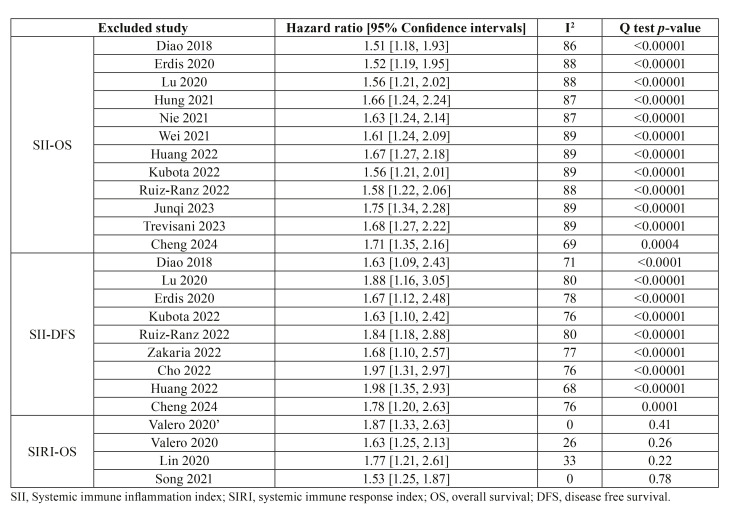
Meta-analysis examining the association between SIRI and OS after oral cancer.

Meta-analysis was possible only for OS for SIRI. Pooled analysis of four cohorts showed that high SIRI was associated with worse OS in oral cancer patients (HR: 1.60 95% 1.31, 1.94) (Fig. 4). We did not note any inter-study heterogeneity in the meta-analysis (I2=0% Q test *p*=0.39).

- Sensitivity analysis

Details of sensitivity analysis are shown in Table 3. On the sequential exclusion of studies, we found that the significance of the results did not change for the association between SII and OS as well as DFS. For OS, the HR varied from 1.51 to 1.75 while for DFS it ranged from 1.63 to 1.98. Likewise, the exclusion of singular cohorts did not change the significance of the association between SIRI and OS. The HR remained statistically significant but ranged from 1.53 to 1.87.

- Subgroup analysis

Subgroup analysis could only be conducted for SII and not SIRI owing to the limited number of studies available for the latter. Results are shown in Table 4. Based on location, a significant association between SII and OS was maintained only for non-Chinese Asian studies but not for Chinese and non-Asian studies. For DFS, results were non-significant for all subgroups based on location. Based on cancer type, SII was found to be a predictor of both OS and DFS in studies on unspecified OSCC and only for DFS in tongue cancer. Subgroup analysis based on sample size (>300 or <300) did not affect OS results but no statistical significance was noted for DFS in studies with larger sample sizes. Based on treatment, we noted a positive association between SII and OS in studies including only surgically treated cases but not those including radiotherapy and chemotherapy as well. On the other hand, both these subgroups demonstrated non-significant results for DFS. Subgroup analysis of both OS and DFS showed that SII cut-offs of >500 were associated with significant results but no such association was noted for cut-off <500. Results were significant only with multivariate analysis and not univariate analysis. The method of determination of cut-off did not affect the results of OS but the ROC curve method was associated with non-significant results for DFS. Results were statistically significant only for studies with moderate risk of bias but not for those with high risk of bias.

## Discussion

In this systematic review, we investigated the role of SII and SIRI in predicting outcomes of oral cancer. A total of 14 studies on SII and three studies on SIRI were available after a detailed literature search. Our results showed that patients with high SII had a statistically significant 1.6 times increased risk of worse OS and DFS as compared to those with lower SII. A combined analysis of four cohorts for SIRI demonstrated a similar 60% increased risk of worse OS in patients with oral cancer. Importantly, the sensitivity analysis demonstrated the robustness of the results as the HR remained above 1.5 and the effect size remained statistically significant on the exclusion of studies one after the other. The lack of publication bias along with the stability of the results on sensitivity analysis lend support to the association between SII/SIRI and outcomes of oral cancer thereby providing quality evidence for clinical practice. Nevertheless, we found large heterogeneity in the meta-analysis of SII which prompted us to conduct a thorough subgroup analysis. We noted that most subgroups based on location, cancer type, sample size, treatment, cut-off, methods of determining cut-off, and analysis method showed statistically significant results except for a few subgroups with a low number of studies. Even when the results of these subgroups turned non-significant, the HR was more >1 and the lower end of 95% CI was very close to 1 indicating a tendency of poor OS/DFS. However, none of the subgroups were able to reduce the inter-study heterogeneity indicating that more intricate variables like characteristics of the study population, stage of cancer, and exact treatment modality are at play and these cannot be thoroughly analyzed without raw data from the included studies.

The outcomes of this review are in agreement with a prior review on this topic. Previously Zhang *et al* (34) in their review of 11 studies have also shown that elevated SII was significantly associated with poor OS (HR: 1.85, 95%CI: 1.48, 2.29) and DFS (HR: 1.77 95%CI: 1.20, 2.61) in patients with oral cancer. Our meta-analysis improves over this prior review by including three recent studies and also examining a similar index, i.e. SIRI which has not been assessed by any other review to date. A large body of evidence shows that both SII and SIRI are important predictors of outcomes in patients with other malignancies. Qiu *et al* (35) in a meta-analysis of eight studies have examined the utility of SII for gastric cancer and found it to predict OS but not DFS. In a pooled analysis of 12 studies, Wang *et al* (36) showed that elevated SII index could predict OS, DFS as well as cancer-specific survival in patients with urinary system cancers. Zeng *et al* (37) in a review of six cohorts found SII to be an independent predictor of OS and progression-free survival in nasopharyngeal carcinoma. Likewise, meta-analytic studies have also provided evidence of the prognostic ability of SIRI. Zhang *et al* (16) combined data from eight studies to show that high SIRI was associated with dismissal OS but not DFS in breast cancer. Ren *et al* (14) in their article included a retrospective cohort of gastric cancer patients and also conducted a meta-analysis of six studies to demonstrate that SIRI was an independent marker of worse OS and DFS in gastric cancer patients.

The ability of both SII and SIRI to predict prognosis is due to their combination of multiple hematological counts which are associated with cancer outcomes. Both indices use baseline levels of neutrophils and lymphocytes but SII additionally uses platelets while SIRI uses monocytes. It has been demonstrated in the literature that a combination of two of these counts in ratios like neutrophil-lymphocyte ratio, platelet-lymphocyte ratio, and lymphocyte-monocyte ratio can also predict outcomes of oral cancers (38). However, combining three instead of two can produce a more robust marker expanding its validity. Individually, all hematological cells have been linked with the pathophysiology of cancer.

Neutrophils are among the hematological cells that can promote tumor angiogenesis, tumor invasion, and metastasis by producing pro-inflammatory cytokines like interleukins, vascular endothelial growth factor, specific proteases (like matrix metalloproteinases and elastases), and chemokines. They can release large quantities of reactive oxygen species, arginase, and nitric oxide causing inactivation of T-cells and reducing the immunity against cancer cells. Further, they can promote cancer progression by multiple mechanisms like aiding the invasive ability of cancer cells and helping them escape immune surveillance via tumor acidosis through the mobilization of H+-pump ATPase (39). On the other hand, lymphocytes form the primary defense mechanism against tumor progression. T lymphocytes encounter tumor antigens and directly kill target cells to produce an anti-tumor effect. B lymphocytes control cancer progression through cytokines like interferon-gamma and tumor necrosis factor-alpha while natural killer cells directly attach malignant tumor cells without the need for antigen activation (40). Platelets can also cause tumor progression by promoting epithelial-mesenchymal transition of cancer cells, improving their motility and resistance to apoptosis, and increasing tumor cell extravasation (34). Lastly, monocytes aid in tumorigenesis by secreting numerous chemokines and cytokines (16). They differentiate into tumor-associated macrophages which can cause apoptosis of CD8+ T cells which have anticancer activity thereby causing tumor progression (16). Given these mechanisms, it can be understood that combining these counts to generate SII and SIRI produces a robust marker that reflects the immune status and inflammatory response of the patient thereby predicting cancer outcomes.

There are certain limitations to our review. First, most studies were from a selected group of countries, and data was not reported from around the world. This prohibits the generalization of evidence. Second, much heterogeneity was noted in the included studies. Differences in cancer location, stage, metastasis status, treatment, and follow-up existed among studies which could have caused the high heterogeneity. Importantly, separate data was not available for different cancer stages and we cannot decipher how these indices behave in early and late-stage cancer. Third, while a large amount of data was available for SII, studies are limited for SIRI and this needs to be addressed by future research. Fourthly, we were only able to assess OS and DFS. Data on disease-specific survival was too scarcely reported for a meta-analysis. Further, we could not compare baseline clinicopathological features of high vs low SII and SIRI groups due to limited reporting by the included studies. Such analysis would have given insights on the baseline features of high SII and SIRI groups and added to the scientific value of the review. Lastly, most data examined in the review was retrospective and hence subject to bias. The quality of included studies was not high and all studies had moderate to high risk of bias.

## Conclusions

Taking into account the limitations of the published studies, SII and SIRI can predict the prognosis of oral cancer. Since both these markers can be easily calculated using routinely available hematological counts, they can aid in rapid prognostication of oral cancer patients with minimal resources. Further research on the utility of these markers for specific oral cancers and cancer stages is needed.

## Figures and Tables

**Table 1:** Details of included studies.

Study		Location	Type of cancer	n	Age (y)	Males	TNM stage	Treatment	Cut-off	Method of cut-off	Out-comes	FU (m)	Analysis
SII	Diao 2018(19)	China	UOSCC	309	NR	171	I-IV	Surgery	484	X-tile	OS, DFS	48	Multi
	Erdis 2020(22)	Turkey	UOSCC	58	67	40	I-IV	RT	954	ROC curve	OS, DFS	1-140	Uni
	Lu 2020(21)	China	Tongue	120	55	79	I-IV	Surgery	569	X-tile	OS, DFS	37.5	Multi
	Hung 2021(25)	Taiwan	UOSCC	993	51	922	I-IV	Surgery+ RT/CCRT	810.6	ROC curve	OS	105.6	Multi
	Nie 2021(24)	China	UOSCC	269	62	204	III-IV	Surgery	535.5	ROC curve	OS	55	Multi
	Wei 2021(23)	China	Tongue	172	69	96	I-IV	Surgery	204	X-tile	OS	65	Uni
	Cho 2022(30)	Korea	UOSCC	269	55	173	I-IV	Surgery	548.9	ROC curve	DFS	1-150	Multi
	Huang 2022(29)	Taiwan	UOSCC	592	54	518	I-IV	Surgery	459	ROC curve	OS, DFS	100	Multi
	Kubota 2022(9)	Japan	UOSCC	183	66	103	I-IV	Surgery+ RT/CCRT	569	Literature	OS, DFS	1-150	Uni-OS; Multi-DFS
	Ruiz-Ranz 2022(28)	Spain	UOSCC	348	62	221	I-IV	Surgery	1137	ROC curve	OS, DFS	54	Uni
	Zakaria 2022(27)	-40	UOSCC	151	59.7	56	I-IV	Surgery+ RT/CCRT	914	ROC curve	DFS	30	Multi
	Junqi 2023(33)	China	Tongue	297	NR	99	I-IV	Surgery	301.5	ROC curve	OS	1-60	Uni
	Trevisani 2023(31)	Brazil	UOSCC	600	61.3	441	I-IV	Surgery+ RT/CCRT	416.1	ROC curve	OS	33.1	Multi
	Cheng 2024(32)	China	Lip	147	NR	71	I-IV	Surgery+ RT/CCRT	NR	NR	OS, DFS	Up to 120	Multi
SIRI	Lin 2020(10)	China	UOSCC	535	NR	424	I-IV	Surgery+ RT/CCRT	1.14	X-tile	OS	Up to 120	Multi
	Valero 2020(20)	Spain	UOSCC	1369	61.9	770	I-IV	Surgery+ RT/CCRT	1	CART method	OS	39	Multi
		Spain	UOSCC	119	66.2	79	I-IV	Surgery+ RT/CCRT	1	CART method	OS	NR	Uni
	Song 2021(26)	China	UOSCC	235	53	128	I-II	Surgery	1.3	X-tile	OS	39	Multi

UOSCC, unspecified oral squamous cell carcinoma; TNM, Tumor, node, metastasis; RT, radiation therapy; CCRT, concurrent chemoradiotherapy; ROC, receiver operating characteristic; OS, overall survival; DFS, disease-free survival; NR, not reported; SII, Systemic immune inflammation index; SIRI, systemic immune response index; Multi, multivariate analysis; Uni, univariate analysis; FU, Follow-up; m, months; y, years; n, number of participants.

**Table 2:** Risk of bias assessment of included studies.

Study		Study participation	Study attrition	Prognostic factor information	Outcome measurement	Study confounding	Statistical analysis and reporting	Risk of bias
SII	Diao 2018(19)	Low	Moderate	Low	Low	Low	Low	Moderate
	Erdis 2020(22)	Low	Moderate	Low	Low	High	Low	Moderate
	Lu 2020(21)	Low	Moderate	Low	Low	Low	Low	Moderate
	Hung 2021(25)	Low	Moderate	Low	Low	Low	Low	Moderate
	Nie 2021(24)	Low	Moderate	Low	Low	Low	Low	Moderate
	Wei 2021(23)	Low	Moderate	Low	Low	High	Low	High
	Cho 2022(30)	Low	Moderate	Low	Low	Low	Low	Moderate
	Huang 2022(29)	Low	Moderate	Low	Low	Low	Low	Moderate
	Kubota 2022(9)	Low	Moderate	Low	Low	Moderate	Low	High
	Ruiz-Ranz 2022(28)	Low	Moderate	Low	Low	High	Low	High
	Zakaria 2022(27)	Low	Moderate	Low	Low	Low	Low	Moderate
	Junqi 2023(33)	Low	Moderate	Low	Low	High	Low	High
	Trevisani 2023(31)	Low	Moderate	Low	Low	Low	Low	Moderate
	Cheng 2024(32)	Low	Moderate	Moderate	Low	Low	Low	High
SIRI	Lin 2020(10)	Low	Moderate	Low	Low	Low	Low	Moderate
	Valero 2020(20)	Low	Moderate	Low	Low	Moderate	Low	High
	Song 2021(26)	Low	Moderate	Low	Low	Low	Low	Moderate

SII, Systemic immune inflammation index; SIRI, systemic immune response index.

**Table 3:** Outcomes of sensitivity analysis.

Excluded study		Hazard ratio [95% Confidence intervals]	I^2^	Q test p-value
SII-OS	Diao 2018	1.51 [1.18, 1.93]	86	<0.00001
	Erdis 2020	1.52 [1.19, 1.95]	88	<0.00001
	Lu 2020	1.56 [1.21, 2.02]	88	<0.00001
	Hung 2021	1.66 [1.24, 2.24]	87	<0.00001
	Nie 2021	1.63 [1.24, 2.14]	87	<0.00001
	Wei 2021	1.61 [1.24, 2.09]	89	<0.00001
	Huang 2022	1.67 [1.27, 2.18]	89	<0.00001
	Kubota 2022	1.56 [1.21, 2.01]	89	<0.00001
	Ruiz-Ranz 2022	1.58 [1.22, 2.06]	88	<0.00001
	Junqi 2023	1.75 [1.34, 2.28]	89	<0.00001
	Trevisani 2023	1.68 [1.27, 2.22]	89	<0.00001
	Cheng 2024	1.71 [1.35, 2.16]	69	0.0004
SII-DFS	Diao 2018	1.63 [1.09, 2.43]	71	<0.0001
	Lu 2020	1.88 [1.16, 3.05]	80	<0.00001
	Erdis 2020	1.67 [1.12, 2.48]	78	<0.00001
	Kubota 2022	1.63 [1.10, 2.42]	76	<0.00001
	Ruiz-Ranz 2022	1.84 [1.18, 2.88]	80	<0.00001
	Zakaria 2022	1.68 [1.10, 2.57]	77	<0.00001
	Cho 2022	1.97 [1.31, 2.97]	76	<0.00001
	Huang 2022	1.98 [1.35, 2.93]	68	<0.00001
	Cheng 2024	1.78 [1.20, 2.63]	76	0.0001
SIRI-OS	Valero 2020'	1.87 [1.33, 2.63]	0	0.41
	Valero 2020	1.63 [1.25, 2.13]	26	0.26
	Lin 2020	1.77 [1.21, 2.61]	33	0.22
	Song 2021	1.53 [1.25, 1.87]	0	0.78

SII, Systemic immune inflammation index; SIRI, systemic immune response index; OS, overall survival; DFS, disease free survival.

**Table 4:** Subgroup analysis for the meta-analysis on SII.

Variable		Groups	Studies	Hazard ratio [95% Confidence intervals]	I^2^	Q test p-value
Overall survival	Location	Chinese	6	1.51 [1.00, 2.27]	89	<0.0001
		Non-Chinese	4	2.00 [1.18, 3.39]	72	<0.0001
		Asian	2	1.53 [0.98, 2.38]	63	<0.0001
	Cancer type	OSCC	8	1.78 [1.40, 2.26]	66	0.0001
		Tongue	3	1.42 [0.58, 3.49]	79	<0.0001
		Lip	1	1.00 [1.00, 1.00]	-
	Sample size	<300	7	1.65 [1.11, 2.47]	86	<0.0001
		>300	5	1.62 [1.24, 2.12]	66	0.003
	Treatment	Surgery	7	1.65 [1.18, 2.31]	70	0.0001
		RT	1	5.76 [2.14, 15.50]	-
		Surgery+ RT/CCRT	4	1.32 [0.97, 1.78]	88	<0.0001
	Cut-off	>500	6	1.94 [1.48, 2.55]	59	0.003
		<500	5	1.39 [0.87, 2.22]	78	0.0001
	Method of cut-off	X-tile	3	2.62 [1.85, 3.70]	0	0.44
		ROC curve	7	1.45 [1.14, 1.85]	68	0.005
		Literature	1	3.28 [1.29, 8.34]	-	-
	Analysis method	Univariate	5	2.00 [1.00, 4.00]	79	0.0001
		Multivariate	7	1.51 [1.15, 2.00]	90	<0.0001
	Risk of bias	Moderate	7	1.76 [1.36, 2.27]	69	0.003
		High	5	1.37 [0.87, 2.16]	79	0.0009
Disease free survival	Location	Chinese	3	1.54 [0.86, 2.76]	93	<0.0001
		Non-Chinese	5	1.84 [0.95, 3.58]	81	<0.0001
		Non-Asian	1	1.48 [0.83, 2.64]	-	-
	Cancer type	OSCC	7	1.88 [1.16, 3.05]	80	<0.0001
		Tongue	1	1.44 [1.01, 2.05]	-	-
		Lip	1	1.00 [1.00, 1.00]	-	-
	Sample size	<300	6	1.66 [1.06, 2.60]	84	<0.0001
		>300	3	1.56 [0.78, 3.14]	87	<0.0001
	Treatment	Surgery	5	1.38 [0.90, 2.11]	78	0.0001
		RT	1	4.60 [1.27, 16.66]	-	-
		Surgery+ RT/CCRT	3	2.03 [0.83, 4.97]	90	<0.0001
	Cut-off	>500	6	1.84 [1.19, 2.84]	64	0.003
		<500	2	1.61 [0.56, 4.63]	93	<0.0001
	Method of cut-off	X-tile	2	1.98 [1.04, 3.76]	82	<0.0001
		ROC curve	5	1.49 [0.89, 2.50]	74	0.005
		Literature	1	4.10 [1.63, 10.31]	-
	Analysis method	Univariate	2	2.24 [0.77, 6.55]	60	0.0001
		Multivariate	7	1.54 [1.06, 2.23]	88	<0.0001
	Risk of bias	Moderate	6	1.67 [1.06, 2.64]	80	0.0001
		High	3	1.59 [0.81, 3.13]	81	0.005

OSCC, oral squamous cell carcinoma; RT, radiation therapy; CCRT, concurrent chemoradiotherapy; ROC, receiver operating characteristic.
